# Possible recent warming hiatus on the northwestern Tibetan Plateau derived from ice core records

**DOI:** 10.1038/srep32813

**Published:** 2016-09-09

**Authors:** Wenling An, Shugui Hou, Wangbin Zhang, Shuangye Wu, Hao Xu, Hongxi Pang, Yetang Wang, Yaping Liu

**Affiliations:** 1 School of Geographic and Oceanographic Sciences, Nanjing University, Nanjing 210093, China; 2 College of Population, Resources and Environment, Shandong Normal University, Jinan 250014, China; 3 State Key Laboratory of Cryospheric Sciences, Cold and Arid Regions Environmental and Engineering Research Institute, Chinese Academy of Sciences, Lanzhou 730000, China; 4 CAS Center for Excellence in Tibetan Plateau Earth Sciences, Beijing 100101, China; 5 Geology Department, University of Dayton, Ohio 45469-2364, USA

## Abstract

Many studies have reported enhanced warming trend on the Tibetan Plateau (TP), even during the warming hiatus period. However, most of these studies are based on instrumental data largely collected from the eastern TP, whereas the temperature trend over the extensive northwestern TP remains uncertain due to few meteorological stations. Here we combined the stable isotopic δ^18^O record of an ice core recovered in 2012 from the Chongce glacier with the δ^18^O records of two other ice cores (i.e., Muztagata and Zangser Kangri) in the same region to establish a regional temperature series for the northwestern TP. The reconstruction shows a significant warming trend with a rate of 0.74 ± 0.12 °C/decade for the period 1970–2000, but a decreasing trend from 2001 to 2012. This is consistent with the reduction of warming rates during the recent decade observed at the only two meteorological stations on the northwestern TP, even though most stations on the eastern TP have shown persistent warming during the same period. Our results suggest a possible recent warming hiatus on the northwestern TP. This could have contributed to the relatively stable status of glaciers in this region.

The Tibetan Plateau (TP) plays an important role in regional and global circulation variations^1^,^2^, owing to its large area and high average altitude of more than 4000 m above sea level (a.s.l.). Because of this importance, the TP’s response to recent climate change has been studied extensively using the meteorological and paleoclimatic records^3^,^4^. However, most previous studies provide an incomplete climate history by either explicitly or implicitly focusing on the middle and eastern TP only. This is largely because climate records from the remote northwestern TP are short and sparse due to its formidable environment and few population^5^,^6^. With the exception for a few studies based on meteorological records^7^, and ice core records^8^,^9^, very little is known about the overall climate change in this region. Since the northwestern TP is an important connection region between the Asian monsoon and middle latitudes^10^,^11^, more high-resolution climate records for this region are needed.

The global average surface temperature has experienced relatively little change since early 2000s, despite the persistent increase in the atmospheric concentration of CO_2_ and other greenhouse gases^12^. Since this recent warming hiatus has been established largely from instrumental records of surface temperature around the world, bias could arise from the uneven spatial coverage^13^, in particular the lack of records in crucial high elevation regions. Based on instrumental records, the period 2001–2012 is the warmest decade for the TP with enhanced warming rate^6^. However, most of the meteorological stations are located on the eastern TP. Only two stations exist on the northwestern TP (Fig. 1), and they show distinctively different temperature trends from that of the eastern TP during the period 2001–2012 (Fig. 1). It is therefore necessary to obtain additional climate information in order to establish a reliable climate history for this crucial region for a better understanding of the behavior of recent warming hiatus over the high elevation regions.

Stable isotopes in high elevation ice cores provide a wealth of climate information that extends beyond the instrumental period, making them a valuable tool for interpreting climate trends on the TP^14^. Several studies have examined the temperature effect on stable oxygen isotopic composition (δ^18^O) in precipitation and ice core for the western TP, and found significant positive correlations between δ^18^O in precipitation and ice core and local temperature^9^,^15^,^16^, such as the isotopic dependence on temperature of Muztagata ice core^9^, and the evident positive correlations between δ^18^O of precipitation and air temperature at the Shiquanhe station^16^. In the light of these studies, we present a high-resolution isotopic record from a new ice core recently drilled on the Chongce glacier (58.82 m in length, 35°14′N, 81°07′E, 6010 m above sea level (a.s.l.)), northwestern TP (Fig. 1). This ice core δ^18^O record is then compared with nearby instrumental records to verify the climate signals of the isotopic record. It is then combined with two previously published ice core δ^18^O records (Muztagata^9^ (38°17′N, 75°06′E, 7010 m a.s.l.) and Zangser Kangri^17^ (34°18′N, 85°51′E, 6226 m a.s.l.), Fig. 1) to reconstruct a regional temperature record in order to better understand past climate change on the northwestern TP.

## Results and Discussion

### The Chongce ice core δ^18^O variation and climatic significance

The δ^18^O of the Chongce ice core varies from −17.47‰ at 5.41 m depth to −5.24‰ at 9.82 m depth, with an average value of −10.31‰ (Fig. S1). This is generally consistent with previous studies^15^ of this region. The δ^18^O series decreases since the 1950s, and stays relatively low from the mid 1960s to the late 1980s (Fig. 2a). Since the 1990s, the δ^18^O value increases significantly until 2008, but decrease sharply from 2009 to 2012 (Fig. 2a).

The stable oxygen isotope composition in precipitation is known to be related to the local temperature^18^. Previous studies have suggested that air temperature is positively related to the δ^18^O in precipitation for the western TP^19^,^20^. The Chongce annual δ^18^O values exhibit significant positive correlation with annual temperature record from the nearby Shiquanhe station (*r *=* *0.43, *n *=* *52, *p *=* *0.002). The correlation becomes more significant (*r *=* *0.87, *n *=* *52, *p *<* *0.001) after Fourier smoothing based on fast Fourier transform algorithm (FFT). The results indicate that the δ^18^O record of the Chongce ice core is a good proxy for the regional annual temperature variations. The instrumental temperature and the ice core δ^18^O records show good agreement, with high values in the early 1950s and a rapid increasing trend from 1990s (Fig. 2d).

Beside temperature, the δ^18^O in precipitation could also be influenced by other factors such as precipitation amount^21^ and seasonality^22^. Based on data from the closest meteorological station (Shiquanhe), summer precipitation accounts for about 84% of the total annual precipitation (Fig. S2a). The percentages of the summer and winter precipitation of the annual precipitation show no significant trend from 1961 to 2012 (Fig. S2b,c). Accumulation rate derived from Chongce ice core from 1953 to 2012 also shows no significant trend (Fig. S2d). In addition, the annual δ^18^O is not significantly correlated with precipitation amount data collected at the Shiquanhe station (*r* = −0.02, *n *=* *52, *p *>* *0.1). The correlation does not improve after FFT smoothing. Therefore, it is unlikely that changes in precipitation amount and seasonality would have a significant influence on δ^18^O variations.

### Regional temperature reconstruction

The Chongce δ^18^O series is compared with two other ice core δ^18^O records (i.e., Muztagata^9^ and Zangser Kangri^17^) from the northwestern TP. All three δ^18^O records show similar temporal patterns, such as the low values during the 1960s and the increasing trend since 1970s (Fig. 2). Further analysis shows significant correlations among these ice core δ^18^O series, particularly after FFT smoothing (Table S1). The correlations between Muztagata and Chongce (*r *=* *0.29, *p *<* *0.05), and Zangser Kangri (*r *=* *0.29, *p *<* *0.05) were slightly lower than that between Chongce and Zangser Kangri (*r *=* *0.39, *p *<* *0.05), due to its greater distance from the other two glaciers. However, the regional composite with Muztagata records correlates very strongly with that without Muztagata (*r *=* *0.85, 1955–2002, *p *<* *0.001), and two series showed very similar temporal patterns (Fig. S3). Therefore, we decided to include the Muztagata ice core δ^18^O, so that the final composite could have a larger spatial coverage to better represent the regional temperature change of the northwestern TP.

Based on this consistency, we establish a regional composite temperature series by using the average of the three ice core δ^18^O records covering their common temporal range (i.e., 1955–2002). In order to make use of the extra years (2003–2012) provided by the Chongce core, we extrapolate the regional series using the linear regression between the Chongce δ^18^O and the regional δ^18^O average values, as the two series are highly correlated (*r *=* *0.71, *n *=* *48, *p *<* *0.001). The regional δ^18^O series shows a significantly positive correlation with the average regional instrumental temperature (i.e., the mean of annual temperature series of Shiquanhe and Taxkorgen stations) (*r *=* *0.57, *n *=* *52, *p *<* *0.001). The correlation improves after FFT smoothing (*r *=* *0.80, *n *=* *52, *p *<* *0.001) for the period 1961–2012, as smoothing could reduce the impact of uncertainty in ice core dating (±1a). Therefore, subsequent analyses are conducted on smoothed values. The correlation result suggests that the regional δ^18^O values are able to capture the overall characteristic of the regional temperature variations.

We further conduct a regression analysis to examine the isotope sensitivity to temperature. It results in the following regression function between the FFT smoothed values of the regional ice core δ^18^O and instrumental temperature anomalies: δ^18^O (‰)* *=* *1.83T (°C) −0.03 (*r *=* *0.80). This isotope–temperature conversion rate is relatively high, but comparable with those reported on the TP^9^,^17^,^23^. Therefore, we use the temperature gradient of 1.83‰ °C^−1^ to convert *δ*^18^O to temperature anomalies for the regional reconstruction on the northwestern TP.

The regional temperature reconstruction shows a relatively cool period during the 1960s, followed by an evident warming trend from the beginning of 1970s to 1998. Since then, it shows a slight decline until 2008 and a sharp decrease from 2009 to 2012 (Fig. 2d). The reconstructed temperature series is largely consistent with the instrumental temperature record for the period from 1961 to 2000 (*r *=* *0.62, *n *=* *40, *p *<* *0.001), but shows great discrepancy for the period from 2001 to 2012 (*r *=* *−0.16, *n *=* *12, *p *>* *0.1) (Fig. 2d). Such a discrepancy could be partially attributed to the large distance and elevation difference between ice core sites in the high mountainous region and the meteorological stations.

### Temperature trends of the northwestern TP

We calculate the linear temperature trends for the three distinct periods: 1970–2012 as a whole, 1970–2000 and 2001–2012, separately. The cut-off years are chosen due to the following reasons: (1) the year 1970 is generally considered as the beginning of a rapid warming trend on the TP^17^,^23^; (2) 2001 is used as the start of the global warming hiatus period^6^. Although in previous studies, years 1998, 2000, 2001, 2002 have been used to indicate the beginning of the global warming hiatus^6^,^12^,^24^,^25^, we choose 2001 to avoid the influence of the extreme high record in 1998 on trend analysis. The use of these commonly recognized years facilitates comparison of our work with previous studies.

The regional temperature reconstruction showed a significant warming trend at a rate of 0.33 ± 0.09 °C/decade for the whole period 1970–2012 (Fig. 3a). The temperature is increasing at a higher rate for the period 1970–2000 (0.74 ± 0.12 °C/decade), but shows a declining trend for the period 2001–2012 (−1.24 ± 0.25 °C/decade). This might imply a possible halt of warming on the northwestern TP during the recent decade (Fig. 3a,g). This decline in the recent decade is consistent with the observed global and China’s temperature trends in the same period. Our analyses show that the change rate of global annual temperature is −0.006 ± 0.04 °C/decade from 2001 to 2012 (Fig. 3f,l). Similar reduction of warming trend is also present for the temperature of whole China at the rate of −0.21 ± 0.24 °C/decade from 2001 to 2012 (Fig. 3e,k).

**Figure 1:**
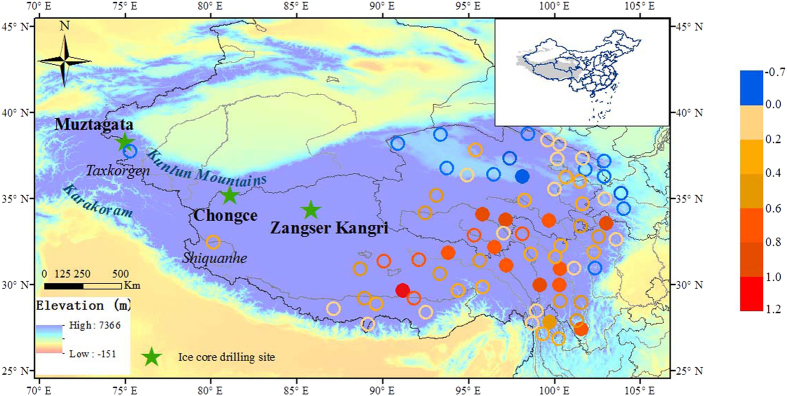
Location of the ice core drilling sites and the spatial distribution of linear trend from meteorological stations on the TP from 2001 to 2012. Map showing the position of Chongce ice core site, two other ice cores sites of Zangser Kangri^17^ and Muztagata^9^ (green star), and meteorological stations on the TP. The circles indicate the linear trend of annual mean temperature series (°C/decade) during 2001–2012. Filled circles indicate the trends are statistically significant at 90% confidence level based on two-tailed Student’s *t* test. Open circles indicate the trends are not statistically significant. The values of linear trend were calculated in Microsoft Excel 2003 and imported into the final map created in Arcmap 10.2 (http://www.esri.com/software/arcgis/). *Scientific Reports* remains neutral with regard to jurisdictional claims in published maps.

**Figure 2:**
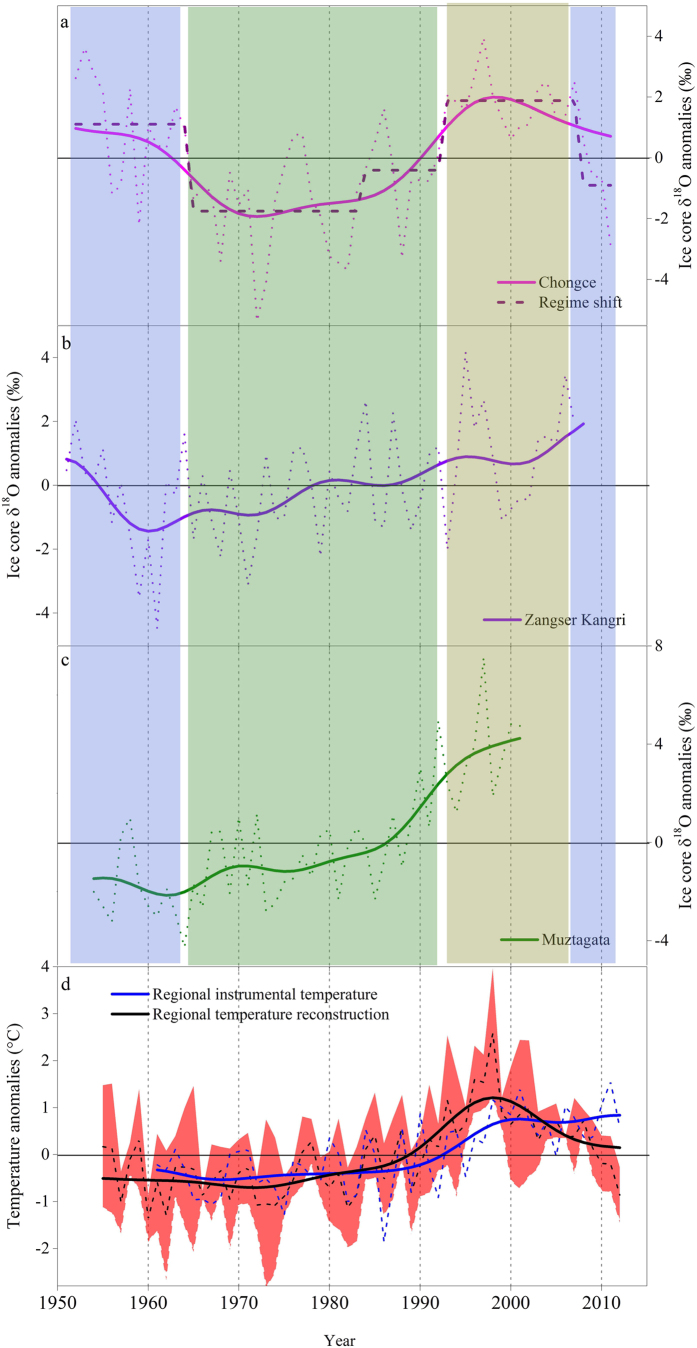
Comparisons of δ^18^O in ice cores from the northwestern TP, and the regional temperature reconstruction series. δ^18^O anomalies in the ice core from Chongce (**a**), Zangser Kangri (**b**) and Muztagata (**c**). Grey dashed lines represent annual values, and solid lines represent the FFT smoothed values. The red dashed line in (**a**) represents the regime shifts (cutoff length = 15 years, values outside the 95% confidence interval). (**d**) Comparison of the regional instrumental temperature (blue, 1961–2012) with the ice core reconstructed regional temperature (black, 1955–2012) for the northwestern TP. The dashed and solid lines in (**d**) represent the raw values and FFT smoothed values respectively. The red shadow area in (**d**) indicates the range of one standard deviation from the mean of reconstructed temperature values.

**Figure 3:**
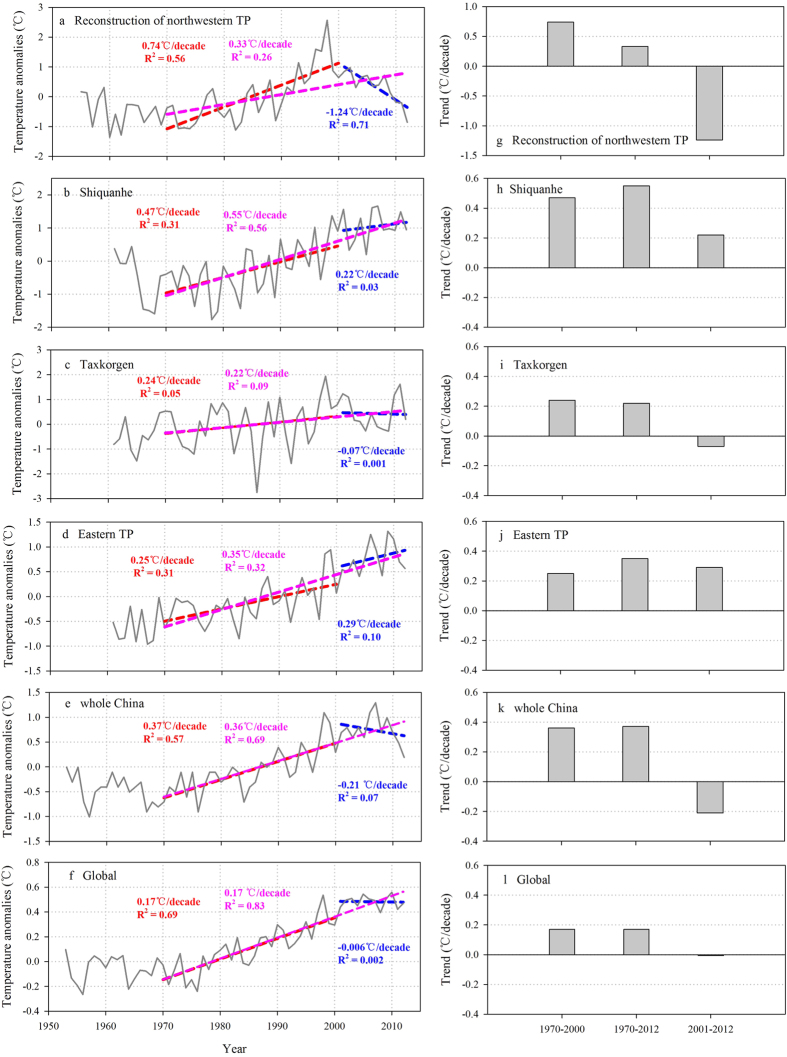
Linear trends of temperature series for the periods It should be ‘1970-2000,1970-2012’ and 2001–2012. Figures on the left show the linear trends of the regional temperature reconstruction of the northwestern TP (**a**), the annual temperature series of Shiquanhe (**b**) and Taxkorgen (**c**) stations from the northwestern TP, temperature series based on all other stations from the eastern TP (**d**), as well as temperature series of China (**e**) and global temperature (**f**), for the periods 1970–2000 (red), 1970–2012 (pink) and 2001–2012 (blue) respectively. The color numbers represent linear trend values (°C/decade) and determination coefficients of the linear fits. Figures on the right (**g**–**l**) shows the bar charts of linear trends (°C/decade) for the three time periods corresponding to above temperature series.

Previous studies suggested enhanced recent warming on the TP despite the reduced warming globally^6^,^24^. These studies were based on the instrumental records collected from the meteorological stations on the TP. Nearly all of them are located on the eastern TP, and only two stations (i.e., Shiquanhe and Taxkorgen) are located on the northwestern TP (Fig. 1). Our analyses show that annual mean temperature series based on the eastern TP stations exhibits significant warming trends for all the three periods: 1970–2012 (0.35 ± 0.04 °C/decade), 1970–2000 (0.25 ± 0.07 °C/decade) and 2001–2012 (0.29 ± 0.27 °C/decade) (Fig. 3d,j). This is consistent with results of previous studies. However, the two stations on the northwestern TP have different temperature patterns. Despite high warming rates for the whole period 1970–2012 (0.55 ± 0.08 °C/decade for Shiquanhe and 0.22 ± 0.11 °C/decade for Taxkorgen, respectively) and the period 1970–2000 (0.47 ± 0.13 °C/decade for Shiquanhe and 0.24 ± 0.19 °C/decade for Taxkorgen, respectively) (Fig. 3b,h,c,i), they show either a significant reduction of warming (0.22 ± 0.43 °C/decade for Shiquanhe) or even a slight cooling trend (−0.07 ± 0.59 °C/decade for Taxkorgen) for the period 2001–2012.

The limited meteorological data from the northwestern TP increase uncertainty for the regional temperature history. In order to further corroborate the temperature trend observed in the ice core reconstruction, we compared it with the regional average temperature series developed from six reanalysis data sets (2° GISS, 0.5° CRU, 0.75° ERA-Interim, 0.5° CPC 2 m, 0.5° UDEL, 2.5° NCEP) extracted for the domain (34–38°N, 75–86°E). The temperature reconstruction shows significant positive correlations with the GISS, CRU, ERA-Interim and the CPC records, but weak correlations with the NCEP 500 hPa and 2 m temperature records (Table S2). These results are not surprising as previous studies have shown that the CRU^26^ and the ERA-Interim^27^,^28^ outperform NCEP in capturing surface temperature variations over the TP. With the exception of NCEP, all other reanalysis temperature data show a decreasing trend during the recent decade (Fig. S4i), confirming a possible recent warming hiatus on the northwestern TP (Fig. 3a,g).

Such a reduction of warming observed in our data, however, cannot easily explained by exact physical mechanisms. Many physical processes have been proposed to explain the recent warming hiatus on the global scale, such as the internal decadal variability of the sea surface temperature (SST) over the equatorial Pacific and North Atlantic oceans 25,29. Meanwhile, the changes in aerosol forcing^30^ and atmospheric circulation^31^ could have caused the recent hiatus for China. As to the northwestern TP, regional processes, e.g., snow-albedo feedback, could play an important role, especially for regions higher than 5000 m that are extensively covered by snow and ice^32^. Ghatak *et al*.^33^ found an increase in snow cover and snow albedo on the western TP during the early 2000s, which may partially contribute to the reduction of warming rate on the northwestern TP.

### Possible links to glacial changes on the northwestern TP

Temperature is one of the most important factors controlling the glacier variations. Glacier change on the TP showed distinct regional variations^2^. From 1970s to 2000s, the most significant glacier shrinkage occurred in the northeastern TP (29.64% for the Qilian Mountains)^34^, followed by the southeastern TP (19.87%) and the Himalayas (16.60% for the Xixiabangma region and 15.63% for Mt. Qomolangma)^2^. On the other hand, glaciers on the northwestern TP showed weak reductions, e.g., 0.4% for the western Kunlun^35^, 1.11% for Muztagata^2^, 0.14% for Karakoram^36^ from 1960s/1970s to 2000s, 0.54% for the eastern Pamirs during the period 2000–2011^37^, and 1.28% for Chongce during the period 1999–2011^38^. In particular, glaciers over Karakoram have even experienced a slight mass gain (+0.11 ± 0.22 m yr^−1^)^39^ during the period 1999–2008, a phenomenon often referred to as Karakoram anomaly. In addition to the possible contribution from more accumulation as a result of increased winter precipitation^40^, the relative stability of the glaciers on the northwestern TP could be partly due to the recent reduction of warming rates as indicated by our regional temperature reconstruction. This could be helpful to stabilize water supplies to major rivers^1^ originated from this region in the near future.

In conclusion, we established a regional temperature reconstruction (1955–2012) based on three ice core stable isotopic records from the northwestern TP. The reconstruction captures the significant warming trend since 1970s and a warming reduction since early 2000s. This could contribute to the glacial stability in this region. However, more high-resolution records are necessary for a better understanding of the regional climate change on the northwestern TP.

## Methods

### Ice core drilling and experiment

The Chongce glacier is located at the western Kunlun on the northwestern TP (Fig. 1), with a snowline altitude about 5900 m a.s.l.^41^. It is 28.7 km in length, covering an area of 163.06 km^2^, with a volume of 38.16 km^3^ (ref. 41). In October 2012, three ice cores were recovered from the glacier (35°14′N, 81°07′E, 6010 m a.s.l., Fig. 1) with the following length: 133.83 m, 135.81 m and 58.82 m. Although it was suggested glaciers in the southern and central TP (e.g. Mt. Nyainqêntanglha and Mt. Geladaindong) could experience dramatic mass loss even at high elevations, probably up to about 5800 m a.s.l.^42^, no obvious melting layer was observed in the Chongce ice core due to its high elevation (6010 m a.s.l.). In fact, the top firn layer is about 40 cm when drilling the Chongce ice core, and firn layers can be found as deep as 12.1 m along the core. The ice cores were kept frozen and transported to the Key Laboratory of Coast and Island development of Ministry of Education, Nanjing University. This study is based on the 58.82 m core. The core was split axially into two halves. One half was stored for archive, and the other half was cut into 1956 samples with intervals of 2~3 cm in a cold room (−20 °C) for analyses of stable isotopes and *β*-activity. The δ^18^O was measured using a Picarro Wavelength Scanned Cavity Ring-Down Spectrometer (WS-CRDS, model: L 2120-i, precision at ±0.1‰). The *β*-activity was measured using Alpha-Beta Multidetector (Mini 20, Eurisys Mesures).

### Ice core dating

On the northwestern TP, the δ^18^O values in modern precipitation show marked seasonal pattern with high values in summer and low values in winter^20^. This seasonality has been previously used in the ice core dating^9^,^15^. The Chongce ice core was dated by counting the annual δ^18^O cycles (Fig. S1). The dating result was verified by a reference of *β*-activity peak in 1963 due to thermonuclear bomb testing, and a second *β*-activity peak corresponding to the 1986 Chernobyl nuclear accident (Fig. S1). Both peaks were also observed in Muztagata ice core^43^. So far, this core was dated back only to 1953 at the depth of 10.03 m, with an estimated uncertainty of ±1 year (Fig. S1). The calculated mean annual accumulation rate is 146.05 mm water equivalent for the period 1953–2012.

### Meteorological data

The station-based monthly mean temperature data were obtained from the China Homogenized Historical Temperature Dataset (1961–2012). The data have been processed to ensure good consistent quality^44^, and are widely used^45^. We selected 73 meteorological stations located on the TP (Fig. 1). Among them, only two stations are located on the northwestern TP: Shiquanhe, 32°30′N, 80°05′E, 4278 m a.s.l., and Taxkorgen, 37°47′N, 75°14′E, 3100 m a.s.l. (Fig. 1). Shiquanhe is the closest station to the Chongce glacier, and its data is used for calibration to the Chongce δ^18^O record. The regional instrumental temperature series on the northwestern TP was established by calculating the averages of the temperature records from the Shiquanhe and the Taxkorgen stations.

The annual mean temperature of the entire China was obtained from China’s climate change monitoring bulletin released by the China Meteorological Administration in 2015. The global annual mean temperature was obtained from the Climatic Research Unit at the University of East Anglia (HadCRU4, ref. 46). In addition, we extracted from GISS (2° × 2°), CRU (0.5° × 0.5°), ERA-Interim 2 m (0.75° × 0.75°), CPC 2 m (0.5° × 0.5°), UDEL (0.5° × 0.5°), NCEP 500 hPa and 2 m (2.5° × 2.5°) temperature data for the northwestern TP (34–38°N, 75–86°E) for comparison with the ice core reconstruction. It is worth noting these data sets all have different data sources and compilation methods. CRU gridded data was created by interpolating available station observations^47^. The NCEP/NCAR reanalysis is a continually updated gridded dataset incorporating observations with numerical weather prediction model output^25^,^48^. The ERA-Interim data include satellite-borne instruments, observations from aircraft, ocean-buoys, radiosonde and other surface platforms^49^. The GISS data is compiled by other groups from measurements at meteorological stations and satellite measurements of ocean surface temperature^50^. The CPC data is a combination of two large individual data sets of station observations collected from the Global Historical Climatology Network version 2 and the Climate Anomaly Monitoring System (GHCN + CAMS)^51^. The UDEL gridded data was estimated from monthly weather-station averages using a combination of spatial interpolation methods such as digital-elevation-model assisted interpolation^52^, traditional interpolation^53^, and climatologically aided interpolation^54^.

### Statistical analysis

We use the Regime shift detection v3.2 software^55^ and the independent samples *t* test method to detect climate regime shifts. The Pearson’s correlation coefficient (*r*) is used to assess the relationship between the Chongce δ^18^O series and the temperature record from the Shiquanhe station. For comparison, we first calculated the δ^18^O anomalies for each ice core series to exclude the difference in the absolute values caused by elevation and distance. To evaluate coherence between the different ice core series, we calculated Pearson’s *r* of the raw and smoothed values based on the Fast Fourier Transform (FFT) method. We reconstructed a regional temperature series for the northwestern TP based on a liner calibration between the FFT smoothed values of the regional ice core δ^18^O series and the regional instrumental temperature record.

## Additional Information

**How to cite this article**: An, W. *et al*. Possible recent warming hiatus on the northwestern Tibetan Plateau derived from ice core records. *Sci. Rep.* **6**, 32813; doi: 10.1038/srep32813 (2016).

## Supplementary Material

Supplementary Information

## References

[b1] Immerzeel W. W., Beek L. P. H. & Bierkens M. F. P. Climate change will affect the Asian water towers. Science 328, 1382–1385 (2010). 20538947 10.1126/science.1183188

[b2] Yao T. . Different glacier status with atmospheric circulations in Tibetan Plateau and surroundings. Nature Clim. Change 2(9), 663–667 (2012).

[b3] Liu X. D. & Chen B. D. Climatic warming in the Tibetan Plateau during recent decades. Int. J. Climatol. 20(14), 1729–1742 (2000).

[b4] Yao T. . δ^18^O records from Tibetan ice cores reveal differences in climatic changes. Ann. Glaciol. 43(1), 1–7 (2006).

[b5] Qin J., Yang K., Liang S. & Guo X. The altitudinal dependence of recent rapid warming over the Tibetan Plateau. Climatic Change 97, 321–327 (2009).

[b6] Yan L. B. & Liu X. D. Has climatic warming over the Tibetan Plateau paused or continued in recent Years? J. Earth Ocean Atmos. Sci. 1(1), 13–28 (2014).

[b7] Wang S. J., Zhang M. J., Wang B. L., Sun M. P. & Li X. F. Recent changes in daily extremes of temperature and precipitation over the western Tibetan Plateau, 1973–2011. Quatern. Int. 313–314, 110–117 (2013).

[b8] Han J. K. . Dating of a shallow ice core from Chongce ice cap using micro-particle content. J. Glaciol. Geocryol. 27(6), 846–852 (In Chinese) (2007).

[b9] Tian L. D. . Recent rapid warming trend revealed from the isotopic record in Muztagata ice core, eastern Pamirs. J. Geophys. Res. 111, D13103 (2006).

[b10] Zhao H. B. & Moore G. W. K. On the relationship between Tibetan snow cover, the Tibetan Plateau monsoon and the Indian summer monsoon. Geophys. Res. Lett. 31, L14204 (2004).

[b11] Watanabe T. & Yamazaki K. Influence of the anticyclonic anomaly in the subtropical jet over the western Tibetan Plateau on the intraseasonal variability of the summer Asian monsoon in early summer. J. Climate 25, 1291–1303 (2012).

[b12] Meehl G. A., Teng H. & Arblaster J. Climate model simulations of the observed early-2000s hiatus of global warming. Nature Clim. Change 4, 898–902 (2014).

[b13] Cowtan K. & Way R. G. Coverage bias in the HadCRUT4 temperature series and its impact on recent temperature trends. Quart. J. Roy. Meteorol. Soc. 140(683), 1935–1944 (2014).

[b14] Thompson L. G. . A high-resolution millennial record of the South Asian monsoon from Himalayan ice cores. Science 289, 1916–1919 (2000). 10988068 10.1126/science.289.5486.1916

[b15] Liu Y. X. Climatic and environmental information recorded in Chongce ice core on the Tibetan Plateau. Master Thesis, Hunan Normal University (2007).

[b16] Yu W. S., Ma Y. M., Sun W. Z. & Wang Y. Climatic significance of δ^18^O records from precipitation on the western Tibetan Plateau. Chin. Sci. Bull. 54, 2732–2741 (2009).

[b17] An W. L. . Significant recent warming over the northern Tibetan Plateau from ice core δ^18^O records. Clim. Past 12, 1–11 (2016).

[b18] Dansgaard W. Stable isotopes in precipitation. Tellus 16(4), 436–468 (1964).

[b19] Yu W. S. . Relationships between δ^18^O in summer precipitation and temperature and moisture trajectories at Muztagata, western China. Sci. China Ser. D 49(1), 27–35 (2006).

[b20] Yu W. S. . Stable isotope variations in precipitation and moisture trajectories on the western Tibetan Plateau, China. Arct. Antarct. Alp. Res. 39(4), 688–693 (2007).

[b21] Masson-Delmotte V. . GRIP deuterium excess reveals rapid and orbital scale changes in Greenland moisture origin. Science 309, 118–121 (2005). 15994553 10.1126/science.1108575

[b22] Gao J., Risi C., Masson-Delmotte V., He Y. & Xu B. Q. Southern Tibetan Plateau ice core δ^18^O reflects abrupt shifts in atmospheric circulation in the late 1970s. Clim. Dyn. 46(1), 291–302 (2015).

[b23] Kang S. C. . Recent temperature increase recorded in an ice core in the source region of Yangtze River. Chin. Sci. Bull. 52(6), 825–831 (2007).

[b24] Duan A. M. & Xiao Z. X. Does the climate warming hiatus exist over the Tibetan Plateau? Scientific Reports 5, 13711 (2015). 26329678 10.1038/srep13711 PMC4557067

[b25] Kosaka Y. & Xie S. P. Recent global-warming hiatus tied to equatorial Pacific surface cooling. Nature 501, 403–407 (2013). 23995690 10.1038/nature12534

[b26] Ren Y. L., Shi Y. J., Wang J. S., Zhang Y. & Wang S. G. An overview of temperature variations on the Qinghai-Tibetan Plateau in the recent hundred years using UK CRU high resolution grid data. J. Lanzhou University (Natural Sciences) 48(6), 63–68 (In Chinese) (2012).

[b27] You Q. L., Fraedrich K., Ren G. Y., Pepin N. & Kang S. C. Variability of temperature in the Tibetan Plateau based on homogenized surface stations and reanalysis data. Int. J. Climatol. 33, 1337–1347 (2013).

[b28] Gao L., Hao L. & Chen X. W. Evaluation of ERA-Interim monthly temperature data over the Tibetan Plateau. J. Mt. Sci. 11(5), 1154–1168 (2014).

[b29] Chen X. & Tung K. K. Varying planetary heat sink led to global-warming slowdown and acceleration. Science 345, 897–903 (2014). 25146282 10.1126/science.1254937

[b30] Kühn T. . Climate impacts of changing aerosol emissions since 1996. Geophys. Res. Lett. 41, 4711–4718 (2014).

[b31] Li Q. X. . China experiencing the recent warming hiatus. Geophys. Res. Lett. 42, 889–898 (2015).

[b32] Pepin N. C. & Lundquist J. D. Temperature trends at high elevations: Patterns across the globe. Geophys. Res. Lett. 35, 14701 (2008).

[b33] Ghatak D., Sinsky E. & Miller J. Role of snow-albedo feedback in higher elevation warming over the Himalayas, Tibetan Plateau and Central Asia. Environ. Res. Lett. 9, 114008 (2014).

[b34] Wang P. Y. . Glacier changes in the Heihe River Basin over the past 50 years in the context of climate change. Resour. Sci. 33(3), 399–407 (2011).

[b35] Shangguan D. H. . Glacier changes in the west Kunlun Shan from 1970 to 2001 derived from Landsat TM/ETM+ and Chinese glacier inventory data. Ann. Glaciol. 46(1), 204–208 (2007).

[b36] Wei J. F. . Surface-area changes of glaciers in the Tibetan Plateau interior area since the 1970s using recent Landsat images and historical maps. Ann. Glaciol. 55(66), 213–222 (2014).

[b37] Zeng L. The glacier variations research in the eastern Pamirs plateau during the last 40 years. Master Thesis, Lanzhou University (2012).

[b38] Li C. X., Yang T. B. & Tian H. Z. Variation of west Kunlun mountains glacier during 1990–2011. Progress in Geography. 32(4), 548–559 (2013).

[b39] Gardelle J., Berthier E. & Arnaud Y. Slight mass gain of Karakoram glaciers in the early twenty-first century. Nature Geosci. 5, 322–325 (2012). 10.1038/nature11324 22914167

[b40] Kapnick S. B., Delworth T. L., Ashfaq M., Malyshev S. & Milly P. C. D. Snowfall less sensitive to warming in Karakoram than in Himalayas due to a unique seasonal cycle. Nature Geosci. 7, 834–840 (2014).

[b41] Shi Y. . Concise Glacier Inventory of China (in Chinese) (Shanghai Popular Science Press, Shanghai, China, 2008).

[b42] Kang S. . Dramatic loss of glacier accumulation area on the Tibetan Plateau revealed by ice core tritium and mercury records. Cryosphere 9, 1213–1222 (2015).

[b43] Tian L. . Chernobyl nuclear accident revealed from the 7010 m Muztagata ice core record. Chin. Sci. Bull. 52(10), 1436–1439 (2007).

[b44] Li Q. X., Liu X. N., Zhang H. Z., Peterson T. C. & Easterling D. R. Detecting and adjusting temporal inhomogeneity in Chinese mean surface air temperature data. Adv. Atmos. Sci. 21, 260–268 (2004).

[b45] Ren G. Y., Xu M. Z. & Chu Z. Y. Change in surface air temperature over China during 1951–2004. Climatic Environ. Res. 10(4), 717–727 (In Chinese) (2005).

[b46] Brohan P., Kennedy J. J., Harris I., Tett S. F. & Jones P. D. Uncertainty estimates in regional and global observed temperature changes: A new data set from 1850. J. Geophys. Res. 111, D12106 (2006).

[b47] Harris I., Jones P. D., Osborn T. J. & Lister D. H. Updated high-resolution grids of monthly climatic observations—The CRU TS3.10 dataset. Int. J. Climatol. 34, 623–642 (2014).

[b48] Kalnay E. . The NCEP/NCAR 40-year reanalysis project. Bull. Am. Meteorol. Soc. 77, 437–471 (1996).

[b49] Dee D. P. . The ERA-Interim reanalysis: configuration and performance of the data assimilation system. Quart. J. Roy. Meteorol. Soc. 137(656), 553–597 (2011).

[b50] Hansen J., Ruedy R., Sato M. & Lo K. Global surface temperature change. Rev. Geophys. 48, RG4004 (2010).

[b51] Fan Y. & van den Dool H. A global monthly land surface air temperature analysis for 1948-present. J. Geophys. Res. 113, D01103 (2008).

[b52] Willmott C. J. & Matsuura K. Smart interpolation of annually averaged air temperature in the United States. J. Appl. Meteorol. 34(12), 2577–2586 (1995).

[b53] Willmott C. J. . Statistics for the evaluation and comparison of models. J. Geophys. Res. 90(c5), 8995–9005 (1985).

[b54] Willmott C. J. & Robeson S. M. Climatologically aided interpolation (CAI) of terrestrial air temperature. Int. J. Climatol. 15(2), 221–229 (1995).

[b55] Rodionov S. N. Use of prewhitening in climate regime shift detection. Geophys. Res. Lett. 33, L12707 (2006).

