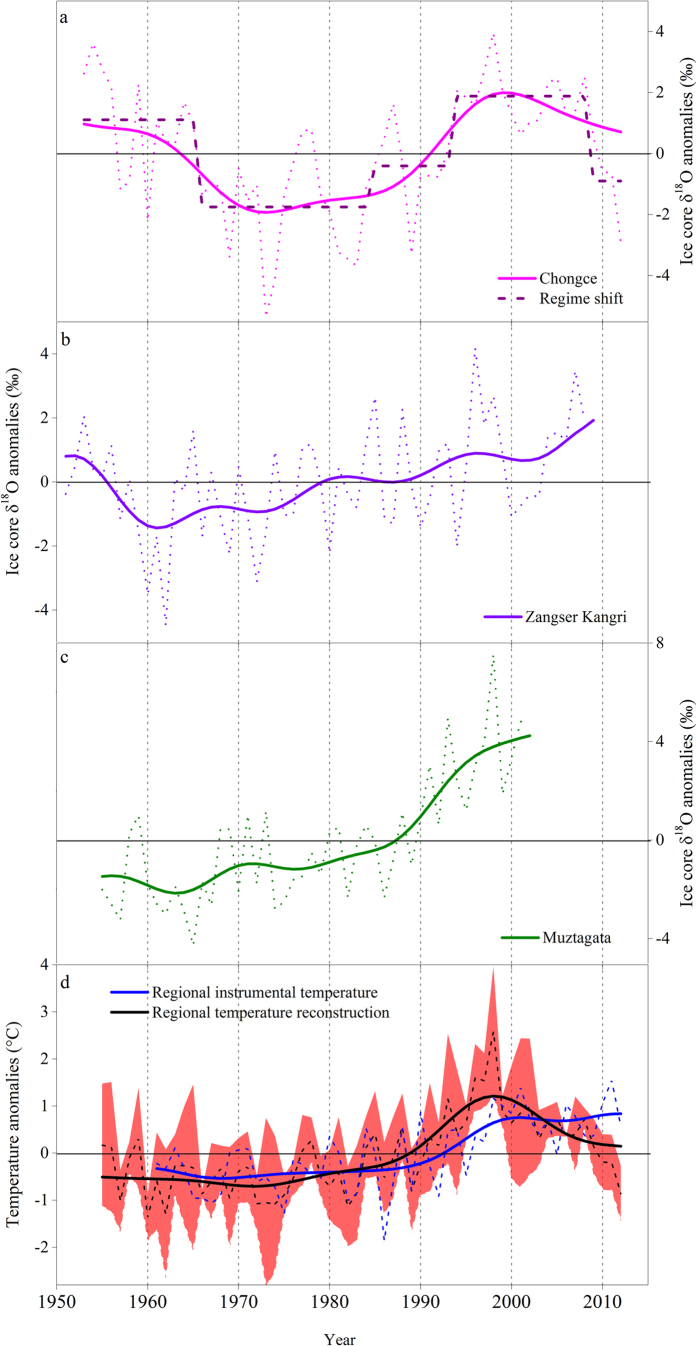# Corrigendum: Possible recent warming hiatus on the northwestern Tibetan Plateau derived from ice core records

**DOI:** 10.1038/srep46863

**Published:** 2017-07-10

**Authors:** Wenling An, Shugui Hou, Wangbin Zhang, Shuangye Wu, Hao Xu, Hongxi Pang, Yetang Wang, Yaping Liu

Scientific Reports 6: Article number: 32813; 10.1038/srep32813 published online: 03 03 2017; updated: 07 10 2017.

This Article contained errors. In the original version, Affiliations 2 and 4 were incorrectly listed as 4 and 2 respectively. The correct affiliations are listed below:

2 CAS Center for Excellence in Tibetan Plateau Earth Sciences, Beijing 100101, China.

4 College of Population, Resources and Environment, Shandong Normal University, Jinan 250014, China.

In addition, the time series for Figure 2 was incorrect and started from 1952. This has been corrected to start from 1951. The correct Figure 2 appears below as Figure 1.

These errors have now been corrected in the PDF and HTML versions of the Article.

## Figures and Tables

**Figure 1:**