# Supplementary material for: Possible recent warming hiatus on the northwestern Tibetan Plateau derived from ice core records
Source: Sci Rep. 2016 Sep 9;6:32813. doi: 10.1038/srep32813 (PMC5017263; doi:10.1038/srep32813)
Supplement: Supplementary Information [file srep32813-s1.doc]

**Possible recent warming hiatus on the northwestern Tibetan Plateau derived from ice core records**

**Wenling An, ^1^ Shugui Hou, ^1, 5*^ Wangbin Zhang, ^1^ Shuangye Wu, ^2^ Hao Xu, ^1^ Hongxi Pang, ^1^ Yetang Wang, ^3^ Yaping Liu ^4^**

1 School of Geographic and Oceanographic Sciences, Nanjing University, Nanjing 210093, China

2 Geology Department, University of Dayton, Ohio 45469-2364, USA

3 College of Population, Resources and Environment, Shandong Normal University, Jinan 250014, China

4 State Key Laboratory of Cryospheric Sciences, Cold and Arid Regions Environmental and Engineering Research Institute, Chinese Academy of Sciences, Lanzhou 730000, China

5 CAS Center for Excellence in Tibetan Plateau Earth Sciences, Beijing 100101, China

* Corresponding author. E-mail: shugui@nju.edu.cn

**Figure S1. Ice core dating.** The variations of δ^18^O in the Chongce ice core and dating results, as well as the total *β*-activity variations with depth.


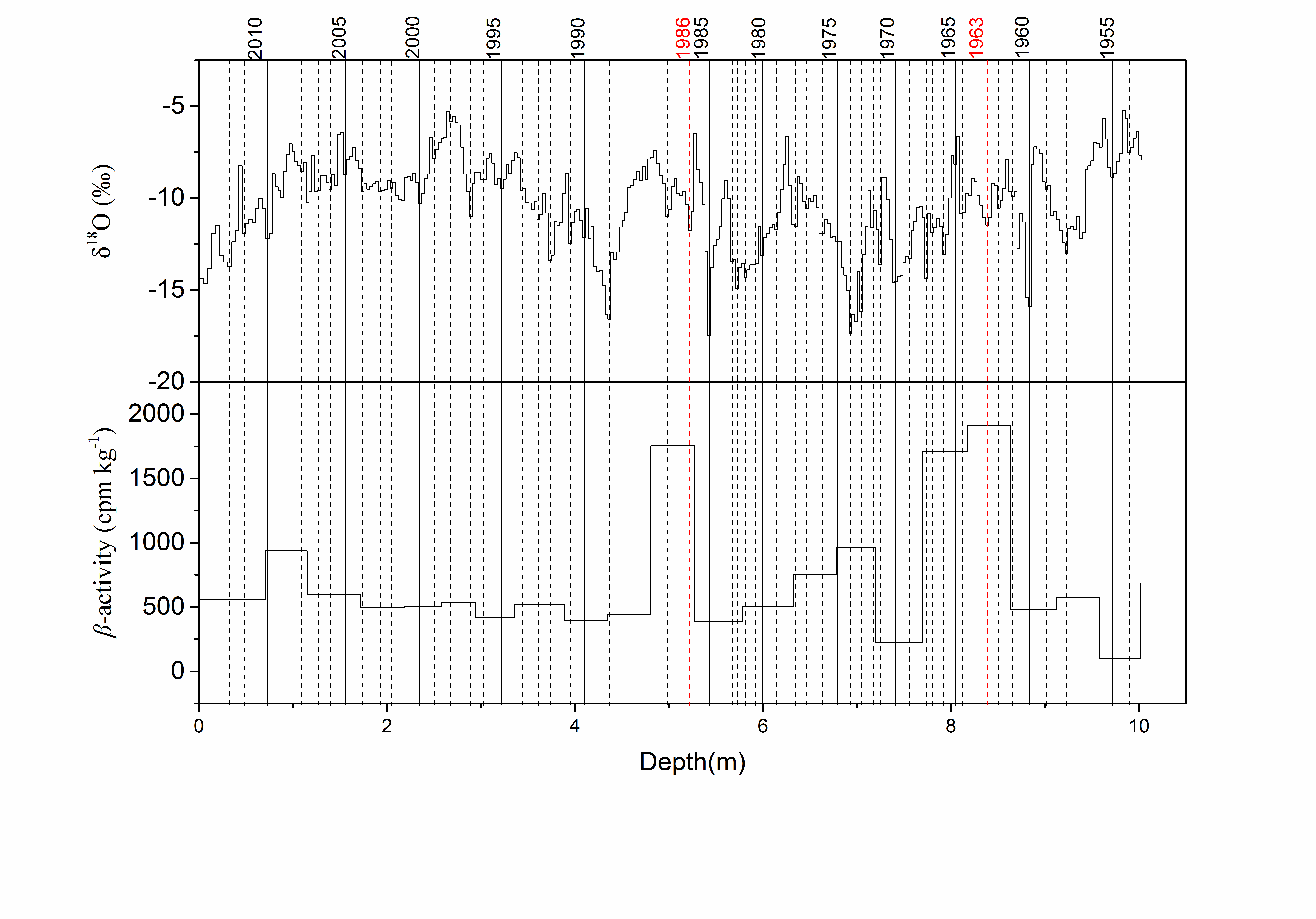


**Figure S2. Observed climate data from the closest meteorological station and accumulation rate from Chongce ice core.** Climatic data from the nearest Shiquanhe meteorological station (Fig. 1) on the northwestern TP: monthly mean temperature (°C) and precipitation (mm) from 1961 to 2012 (a); the percentage of summer (June-September) precipitation (b) and winter (December-February) precipitation (c) in annual precipitation amount from 1961 to 2012; and net accumulation rate of Chongce ice core from 1953 to 2012 (d). Thin and thick solid lines represent the raw and FFT smoothed values, respectively. Dashed lines represent the linear trends.


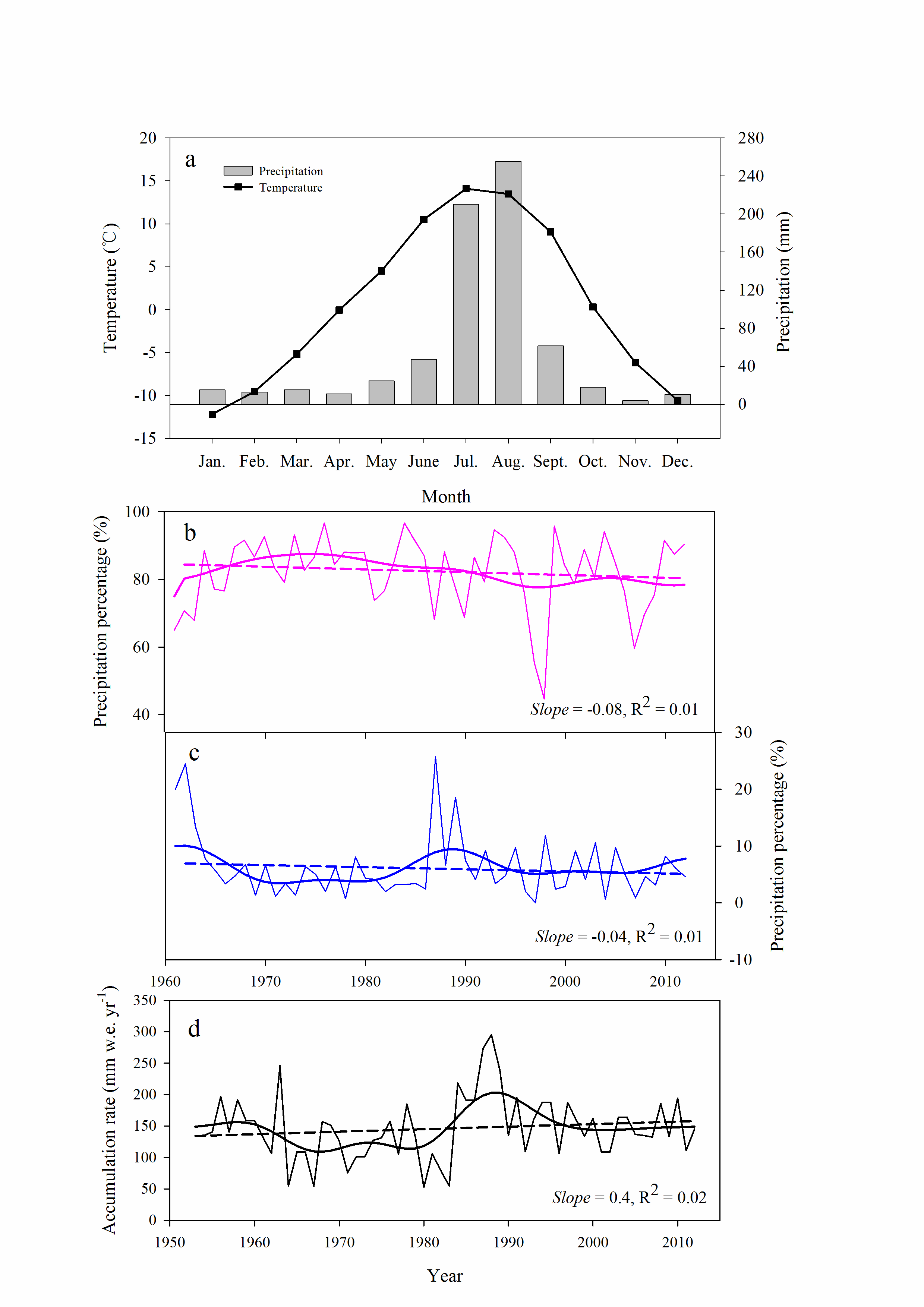


**Figure S3. Comparison between regional temperature reconstruction with and without Muztagata.** The regional ice core δ^18^O time series (from 1955 to 2002) averaged from two ice cores (including Chongce and Zangser Kangri), and from three ice cores (with Muztagata). The shadowed area indicates the range of one standard deviation from the mean.


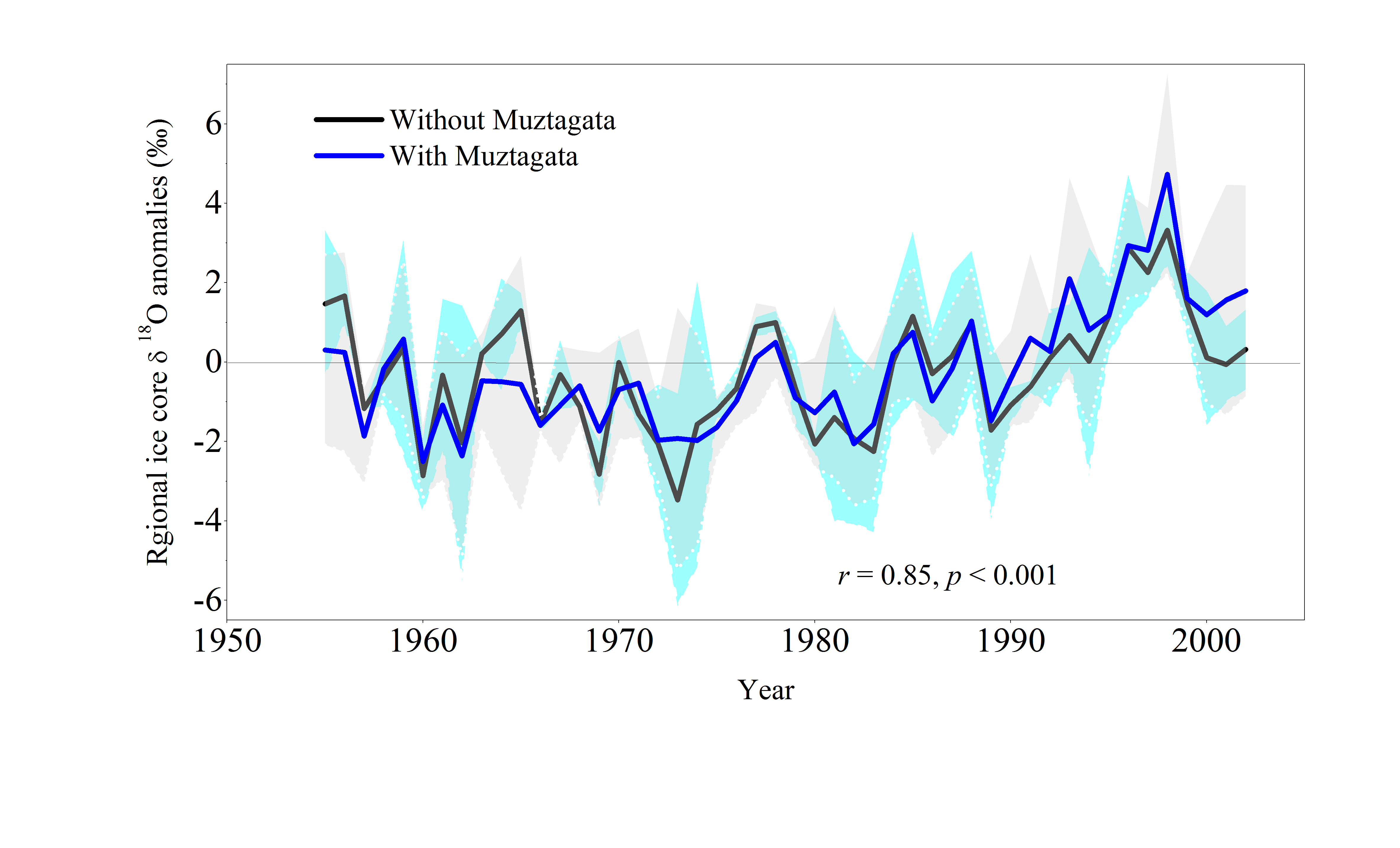


**Figure S4. Comparisons between regional temperature reconstruction of northwestern TP and gridded annual mean temperature anomalies.** Comparisons of regional temperature reconstruction 1955-2012 (a) with instrumental temperature from the 2° GISS 1955-2012 (b), 0.5° CRU 1955-2012 (c), 0.75° ERA-Interim 1979-2012 (d), 0.5° CPC 2 m 1955-2012 (e), 0.5° UDEL 1955-2012 (f), 2.5° NCEP 500 hPa 1955-2012 (g) and 2 m 1955-2012 (h) annual gridded temperature data. The bar charts represent annual values and the solid lines represent the FFT smoothed values. Bar chart in (i) indicates the linear trends (°C/decade) corresponding to these temperature series, * *p* < 0.05, ** *p* < 0.01.


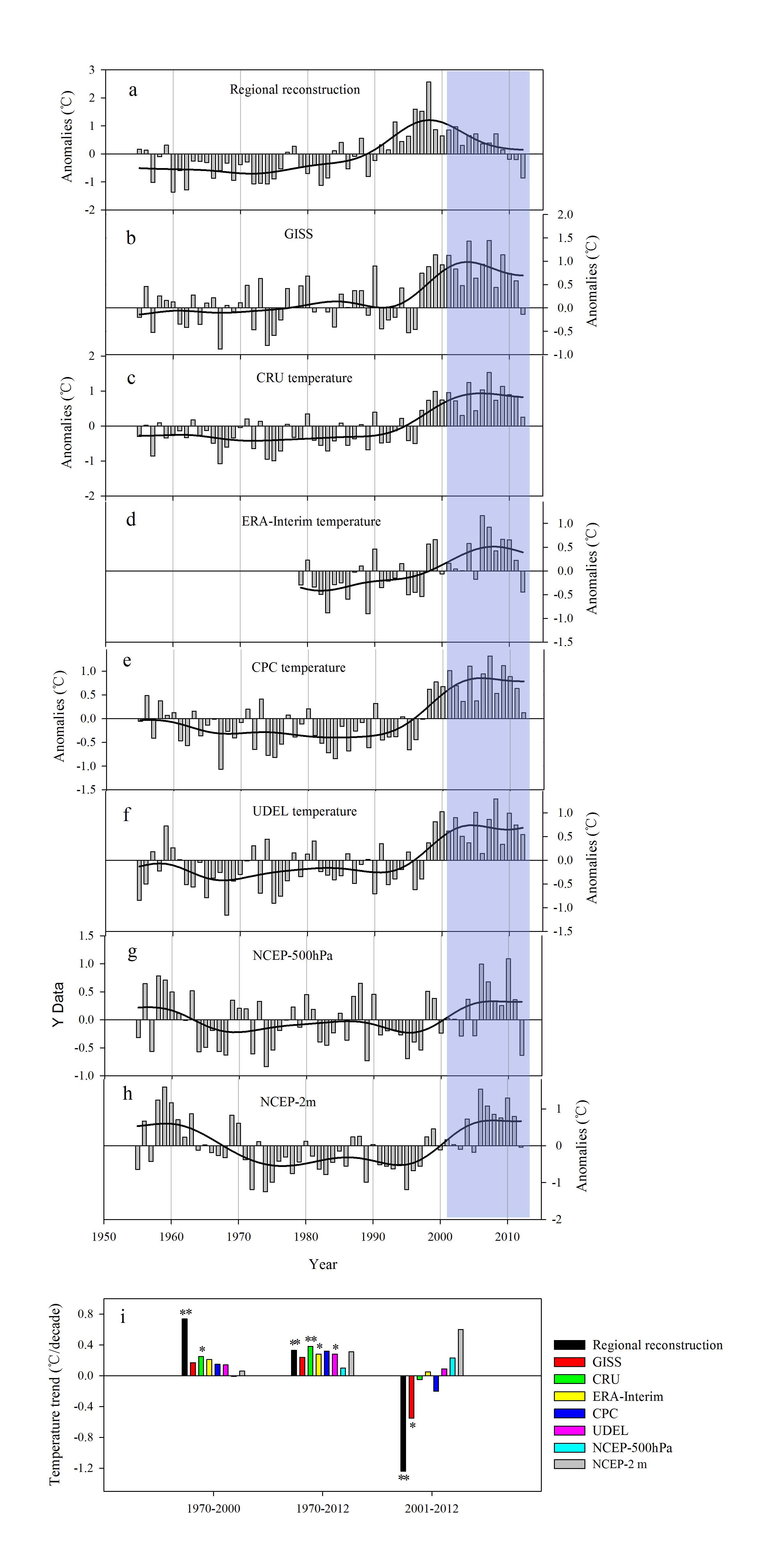


Table S1. Correlation coefficients between the δ^18^O values in the Chongce (1953-2012), Zangser Kangri (1951–2008), Muztagata (1955–2002) ice cores. The values in bold are the correlation coefficients of annual values, and the values in italic are the correlation coefficients of FFT smoothed values.

|  | Chongce | Zangser Kangri | Muztagata |
| --- | --- | --- | --- |
| Chongce |  | **0.39^b^** | **0.29^a^** |
| Zangser Kangri | *0.55 ^b^* |  | **0.29^a^** |
| Muztagata | *0.65 ^b^* | *0.86^b^* |  |

^a^ *p*< 0.05; ^b^ *p*< 0.01

Table S2. Correlation coefficients between regional temperature reconstruction for northwestern TP (1955-2012), and annual temperature series from GISS (1955-2012), CRU (1955-2012), ERA-Interim dataset (1979-2012), CPC (1955-2012), UDEL (1955-2012), NCEP 500 hPa and 2 m (1955-2012).

|  | GISS | CRU | ERA  -Interim | CPC | UDEL | NCEP-  500hPa | NCEP-2m |
| --- | --- | --- | --- | --- | --- | --- | --- |
| Annual value | 0.44 ^b^ | 0.52 ^b^ | 0.30^a^ | 0.42 ^b^ | 0.27 | 0.19 | 0.09 |
| FFT smoothed values | 0.72 ^b^ | 0.71 ^b^ | 0.42^a^ | 0.60 ^b^ | 0.65 ^b^ | 0.09 | 0.005 |

^a^ *p*< 0.1; ^b^ *p*< 0.01
